# Mitigating the effects of climate change on human health with vaccines and vaccinations

**DOI:** 10.3389/fpubh.2023.1252910

**Published:** 2023-10-12

**Authors:** Cara Lynn Kim, Suneth Agampodi, Florian Marks, Jerome H. Kim, Jean-Louis Excler

**Affiliations:** ^1^International Vaccine Institute, Seoul, Republic of Korea; ^2^Section of Infectious Diseases, Department of Internal Medicine, Yale School of Medicine, New Haven, CT, United States; ^3^Cambridge Institute of Therapeutic Immunology and Infectious Disease, University of Cambridge School of Clinical Medicine, Cambridge, United Kingdom; ^4^Madagascar Institute for Vaccine Research, University of Antananarivo, Antananarivo, Madagascar; ^5^Heidelberg Institute of Global Health, University of Heidelberg, Heidelberg, Germany; ^6^College of Natural Sciences, Seoul National University, Seoul, Republic of Korea

**Keywords:** climate change, mitigation, vaccine-preventable diseases, vector-borne diseases, waterborne diseases, vaccine development, supply, delivery

## Abstract

Climate change represents an unprecedented threat to humanity and will be the ultimate challenge of the 21st century. As a public health consequence, the World Health Organization estimates an additional 250,000 deaths annually by 2030, with resource-poor countries being predominantly affected. Although climate change’s direct and indirect consequences on human health are manifold and far from fully explored, a growing body of evidence demonstrates its potential to exacerbate the frequency and spread of transmissible infectious diseases. Effective, high-impact mitigation measures are critical in combating this global crisis. While vaccines and vaccination are among the most cost-effective public health interventions, they have yet to be established as a major strategy in climate change-related health effect mitigation. In this narrative review, we synthesize the available evidence on the effect of climate change on vaccine-preventable diseases. This review examines the direct effect of climate change on water-related diseases such as cholera and other enteropathogens, helminthic infections and leptospirosis. It also explores the effects of rising temperatures on vector-borne diseases like dengue, chikungunya, and malaria, as well as the impact of temperature and humidity on airborne diseases like influenza and respiratory syncytial virus infection. Recent advances in global vaccine development facilitate the use of vaccines and vaccination as a mitigation strategy in the agenda against climate change consequences. A focused evaluation of vaccine research and development, funding, and distribution related to climate change is required.

## 1. Introduction

Climate change is posing an unprecedented threat to humanity. Attributable to human activities and natural climate variability, climate change refers to the long-term changes in weather patterns, and often specifically to the rise in global temperatures. The last decades of progress in global development may be undermined by this crisis. While global temperatures have already seen a human-induced rise of about 1°C since pre-industrial times, the Intergovernmental Panel on Climate Change (IPCC) projects a rise of global temperatures by at least 1.5°C between 2030 and 2052, with a worst-case scenario of up to 5°C increase by 2100 (1, 2). Meanwhile, an estimated 3.3–3.6 billion people already “live in contexts that are highly vulnerable to climate change” (3). While climate change is estimated to have caused over 150,000 deaths globally in 2000, 83 million cumulative additional deaths have been projected by the year 2100 (4, 5). The World Health Organization (WHO) estimates the direct damage costs to health to be around 2–4 billion USD/year by 2030, while others estimate the health-related cost of air pollution and climate change to already have surpassed 800 billion USD/year for the United States alone (6, 7). These widely diverging figures may be attributed to different modeling approaches as well as varying inclusion variables, as the latter figure specifically relates not only to climate-caused, but also climate-sensitive health outcomes. Consequently, the wide range reflects the uncertainties related to accurately describing the effects of climate change.

The main direct and measurable effects of climate change are rising temperatures, extreme weather events such as heat waves, droughts, floods, and changes in precipitation patterns, as well as elevating sea- and greenhouse gas levels (1). These climate hazards can further lead to a cascade of numerous indirect events that may impact human health negatively. Extreme weather events can lead to the displacement of populations, limit access to healthcare services and the availability of clean water and food, thereby changing the social and environmental determinants of health (7, 8).

Climate change is a major driver of infectious disease dynamics through changes in temperature and precipitation. The effect of climate change on infectious diseases disproportionally affects low- and middle-income countries (LMIC) (9). Diseases with a significant global burden are thought to be aggravated by climate change, while the emergence and re-emergence of other infectious diseases are also anticipated (10, 11). A recent study on 375 infectious diseases worldwide found that 58% of them have been exacerbated by climate change through numerous pathways, most notably vector-borne and water-borne diseases and by bringing pathogens closer to people (12). Additionally, climate change is projected to alter the geographic range of mammals as well as humans and thereby facilitate pathogen sharing as there will be an increasing overlap in species range. This could lead to the emergence of zoonotic diseases of pandemic potential; similar to the spillover of HIV and SARS-CoV to humans enabled through wildlife host jumps; further, bats were identified as a major force facilitating future viral sharing (13). Notably, areas of high human population density are anticipated to emerge as future hotspots for these incidents, highlighting the impact of human interference on the alteration of ecological habitats (13, 14).

Mitigating climate-change-related infectious disease threats through climate change mitigation strategies will necessitate considerable long-term efforts and time to achieve impact. While measures to address climate change and its impact currently focus on political, economic, and social strategies, the potential role of vaccines as a strategy to mitigate the consequences of climate change has been poorly explored. The discovery of immunization and the subsequential development of vaccines for previously life-threatening infectious diseases has already saved millions of lives globally. Vaccines have become an indispensable tool in the last decades for preventing and controlling infectious diseases, as recently seen during the COVID-19 pandemic (15, 16). This paper reviews the leading vaccine-preventable infectious diseases associated with climate change, the vaccine development stages for those diseases, and their challenges.

## 2. Climate-sensitive and vaccine-preventable diseases

Almost all pandemic-prone infectious diseases and diseases with high burden are being investigated for vaccines. While vaccines are available to prevent leading childhood infectious diseases, vaccines preventing other infectious diseases with high burden are still to be developed. Table 1 summarizes the climate change-associated vaccine-preventable (or possible) diseases and illustrates the status of vaccine development for those selected conditions.

**Table 1:** Climate change-associated major infectious diseases and corresponding vaccine development status.

Disease	Pathogen	Vector	Non-human reservoir of relevance	Global burden/incidence	Regions with major burden	Examples of observations and projections under climate change	WHO prequalified vaccine (17)	Vaccine candidates and status of development
Food- and Water-Related								
Cholera	*Vibrio cholerae*	n/a	/	1.3–4.0 million (18)	Africa, Asia	Increased environmental suitability Influenced by climatic factors (e.g., temperature, humidity, precipitation) Outbreaks following extreme weather events (19–25)	Euvichol-Plus® Shanchol™ Dukoral®	Several nationally licensed, not WHO-prequalified vaccines available Several preclinical and clinical candidates (26)
Typhoid/Paratyphoid	*S. typhi* *S. paratyphi*	n/a	/	5.9–14.1 million 2.3–6.1 million (27)	Africa, the Americas, South-East Asia, Western Pacific	Influenced by climatic factors (28) Outbreaks following extreme weather events (29)	TypbarTCV® Typhibev® Typhim-Vi®	Several preclinical and clinical candidates (30)
Invasive non-typhoidal salmonella (iNTS)	*S. typhimurium* *S. Enteridis*	n/a	/	0.4–0.7 million (27)	Sub-Saharan Africa	Influenced by climatic factors (31)	n/a	Several candidates at preclinical or early clinical development stage (32)
(Other) Diarrheal Disease	*Enterotoxic E. coli*	n/a	/	~ 145–323 million (33)	Africa, Asia	Influenced by climatic factors (34)	n/a	Several preclinical and clinical candidates (35)
	*Rotavirus*	n/a	/	~ 258 million (children under the age of 5) (36)	Asia, South America	Influenced by climatic factors (37)	Rotarix™ RotaTeq™ Rotavac™ RotaSiil™ Rotavac 5D®	Several preclinical and clinical candidates (38, 39)
Shigellosis	*Shigella*	n/a	/	~ 176–369 million (33)	Africa, Asia, South America	Influenced by climatic factors (40)	n/a	Several preclinical and clinical candidates S. Flexneriza-*S. sonnei* Bivalent Conjugate Vaccine in Phase 3 (41, 42)
Hookworm disease	*Necator americanus* *Ancylostoma duodenale*	n/a	/	n/a, ~ 230 million prevalence (43)	Africa, South America, Asia	Influenced by climatic factors Shift in species distribution (44)	n/a	Na-GST-1/Na-APR-1 in Phase 1 clinical studies (45)
Schistosomiasis	*Schistosoma*	n/a	Snail	n/a, ~ 142 million prevalence (43)	Africa	Influenced by climatic factors Shift in expansion to cooler areas (46, 47)	n/a	Several candidates in preclinical/clinical stages Sh28GST/Bilhvax in Phase 3 (48, 49)
Vector-borne								
Malaria	*Plasmodium* parasite	*Anopheles mosquito*	/	186–290 million (27)	Africa	Northward expansion and lengthened transmission season Regional decreases in endemic areas (50–54)	Mosquirix	R21/Matrix-M in Phase 3 trials (55, 56)
Dengue	Flavivirus	*A. aegypti, A. albopticus*	/	37–101 million (27)	Asia, Americas	Higher suitability in Sub-Sahara Africa compared to Malaria Increased suitability for Europe (57–59)	Dengvaxia®	5 in clinical development TV-003 and TAK-003 in Phase 3 (60, 61)
Zika	Flavivirus	*A. aegypti, A. albopticus*	/	0.2–0.3 million (27)	Africa, Americas, Asia	Lengthened transmissions season Increased risk of transmission globally (62, 63)	n/a	Several in preclinical/Phase 1 VRC-ZKADNA090-00-VP only Phase 2 candidate (64)
Chikungunya	*Alphavirus*	*A. aegypti, A. albopticus*	/	0.69 million (65)	Africa, Asia, Americas	Geographic expansion to Central Europe, China, Central America Declining suitability in other areas (66–68)	n/a	Several in preclinical, Phase 1/2 Valneva VLA1553 completed Phase 3, regulatory ongoing (69)
Yellow Fever	*Flavivirus*	*A. aegypti, A. albopticus*	Non-human primates	0.04–0.24 million (27)	Africa, Central and South America	Heterogenous changes for transmission across African region Varying results of modeling studies for future burden (70, 71)	Stamaril SinSaVac	Several second-generation candidates in preclinical 2 candidates in Phase 1 (72, 73)
Rift Valley Fever	*Bunyaviridiae*	*Aedes, Culex*	Livestock (Cattle, sheep, goats)	n/a	Sub-Saharan Africa	Influenced by climatic factors Geographic expansion (74–76)	n/a	Licensed vaccine for livestock No licensed vaccine for humans, ChAdOx1 candidate in Phase 1 (77)
Lymphatic filariasis	*Wuchereria bancrofti* *Brugia malayi* *B. timor*	*Ae. aegypti, C. quinquefasciatus*	/	~ 51 million (78)	Asia, Africa, Western Pacific, South America	Geographic expansion with shifting patterns of distribution (44, 79, 80)	n/a	Preclinical candidates (81)
Leishmaniasis	*Leishmania*	*Phlebotominae*	Rodents, dog	0.7–1 million (82)	Africa, Asia, Mediterranean, South America	Influenced by climatic factors Geographic expansion (83, 84)	n/a	Several preclinical and clinical candidates (85)
Lyme disease	*Borrelia spirochete*	*Ixodes ticks*	Mouse, small mammals, birds	0.53 million (65)	North America, Europe, Asia	Geographic expansion, esp. northwards and to higher altitudes (86–91)	n/a	VLA15 in Phase 3 LYMERix licensed 1998 (FDA) but withdrawn from market (92)
Tick-borne encephalitis	*Flavivirus*	*Ixodes ticks*	Small rodents	0.01 million (65)	Europe, Asia	Geographic expansion Shift to higher altitudes (93–98)	n/a	FSME-Immun, Encepur, TBE-Moscow, EnceVir (nationally licensed) (99)
Crimean-Congo Hemorrhagic Fever	*Bunyaviridiae*	*Hyalomma ticks*	Wild and domestic animals	n/a	Africa, Balkans, Middle East, Asia	Geographic expansion to Europe Reduced suitability in North Africa and Southern Iberia (100–103)	n/a	Preclinical candidates Vaccine in Bulgaria since 1974 (safety/efficacy concerns) (104)
Air-borne								
Respiratory illness	*Seasonal Influenza Virus*	n/a	Aquatic birds, pigs	3–5 million (105)	Global	Influenced by climatic factors Reduced suitability due to warming climate Increased risk of epidemics/pandemics due to higher weather variability and novel viral pathogens (106–112)	Several licensed flu vaccines available	Yearly adaptations required due to antigenic drift Development of next-generation influenza vaccines ongoing (113)
Respiratory illness	*Respiratory Syncytial Virus*	n/a	n/a	33 million (under 60 months) (114)	Global	Influenced by climatic factors (115–117)	n/a	First RSV vaccine for infants up to 6 months and over 60 years available since 2023, approved by the FDA and recommended by the EMA (118, 119) Several preclinical and clinical candidates (120)
Other								
Leptospirosis	*Leptospira*		Rodents	0.43–1.75 million (43)	South and South-East Asia, Americas, sub-Saharan Africa	Outbreaks following extreme weather events (121, 122)	n/a	Vaccine available for pets Vaccines licensed in France, China, Japan, Cuba Recombinant vaccine in preclinical development (123)

Data sourced from the World Health Organization and US Centers for Disease Control and Prevention.

### 2.1. Food- and water-related diseases

Human health highly depends on access to safe drinking water, sanitation, and hygiene (WASH). WHO estimates 829,000 unsafe WASH-related diarrheal deaths per year, while the disease burden is particularly high in LMICs, where children under 5 years of age are predominantly affected (124). The major WASH-related pathogens are *Vibrio cholera*, *Salmonella Typhi*, and other diarrheal pathogens. Their occurrence has been linked to increasing temperatures and rainfall, thus affected directly by climate change (34, 125–129). The potential effect of extreme weather events exacerbated by climate change on waterborne diseases is becoming an increasing concern: The El Niño-Southern Oscillation (ENSO) is a complex natural and periodic climate pattern that occurs in the Pacific Ocean with extreme phases such as the El Niño and it has shown to be intensified through climate change (130, 131). ENSO causes droughts, floods, and hurricanes worldwide with an enhancement of endemic food- and water-borne diseases, as well as vector-borne diseases, through changes in pathogen survival and proliferation, vector survival, transmission, and ecology (132). Extreme weather events preceding outbreaks of food- and water-borne diseases are reported worldwide, such as typhoons in China, floods in Bangladesh and tsunamis in Thailand and Indonesia (133).

*Cholera* is at the forefront of vaccine-preventable WASH-related diseases, associated directly with climate change. Survival of the *Vibrio cholera* bacterium that causes cholera depends on environmental conditions such as temperature, pH, salinity, and plankton. Increasing sea surface temperature, a well-known impact of climate change, has been found to favor cholera outbreaks (19, 20). In addition, cholera cases have been shown to correlate with rainfall and temperatures while also exhibiting seasonal patterns (21–23). Extreme weather condition-related surges of cholera were documented following El Niño in the African continent, floods in Bangladesh and cyclones in Malawi (22, 24, 25, 134). Most recently, severe droughts in Somalia resulted in large displacement that exacerbated endemic cholera outbreaks. Humanitarian crises like these lead to disrupted health systems, worsened living conditions, limited access to clean water, and malnutrition; all of which may increase susceptibility to pathogens (135).

Predictive models suggest an increase in environmental suitability for *Vibrio cholera* in ocean waters as well as an increase in cholera cases under different Representation Concentration Pathway (RCP) scenarios (136, 137). The RCPs are commonly used for modeling studies and represent four future climate scenarios measured by emissions and classified as RCP 2.6, 4.5, 6.0, and 8.5 with increasing levels of severity, respectively (138). While less extensively than for cholera, similar positive relationships to temperature, rainfall, flooding, and seasonal effects have been reported for other common diarrheal pathogens like *E. coli*, rotavirus, *S. typhi* and other Salmonella (28, 29, 31–34, 37, 139). Pathogen-specific responses to climate variables are likely, as temperature sensitivity has been shown to vary between enteric pathogens (140). For instance, temperature may affect the expression of virulent genes, survival and growth of bacterial pathogens, which exhibit distinct optimal temperatures ranges. Meanwhile, fluctuations in rainfall can shape the ecological niche of enteric pathogens, and lead to contamination of water sources, enhancing their exposure to human populations (129, 140). Unfortunately, most studies do not further differentiate diarrheal diseases according to their causative pathogen (129).

*Schistosomiasis* and *Hookworm* are human helminth infections posing a major health threat to more than a billion people worldwide and are expected to be impacted by climate change. The effects of climate change on water reservoirs directly impact the immediate host of Schistosoma species, poikilothermic freshwater snails (46). Predictions for climate change scenarios suggest a shift in schistosomiasis incidence in African regions where schistosomiasis is currently highly prevalent (47). The burden of soil-transmitted helminthiasis such as hookworm infection, which is known to cause substantial global morbidity, may increase further as climate change impacts soil through increased surface temperatures and altered precipitation (44, 141, 142).

Other water-related zoonotic diseases are also expected to be impacted by climate change. Leptospirosis, the most globally widespread zoonotic disease, is associated with climatic factors. Outbreaks have been reported globally following extreme weather events like flooding (121, 143, 144). Increased rainfall affects environmental conditions for rodent populations, and may further alter transmission dynamics (122, 144).

### 2.2. Vector-borne diseases

The WHO estimates vector-borne diseases to account for 17% of infectious diseases, resulting in more than 700,000 deaths annually. The burden of diseases is highest in low-income settings, with the majority of cases occurring in tropical and subtropical regions (145). Further, the mortality rate from vector-borne diseases is estimated to be almost 300 times greater in LMICs than in high-income countries (146). Temperature significantly influences the survival and transmission of vectors and the pathogens they carry (147). The IPCC states, “risks from some vector-borne diseases, such as malaria and dengue fever, are projected to increase with warming from 1.5°C to 2°C, including potential shifts in their geographic range” (2).

*Malaria* remains one of the most prevalent and fatal infectious diseases worldwide. Nearly half the world’s population is at risk (148). Transmission of the *Plasmodium* parasites occurs through the Anopheles mosquito. Life cycle and disease transmission by *Anopheles gambiae* is influenced strongly by temperature, with an optimal temperature for transmission estimated around 25°C and temperatures between 17°C and 34°C required for survival (149–151). In the past century, Anopheles have shown a gradual movement to higher elevations and greater distances from the equator (50). Global warming is expected to contribute to a further northward expansion of transmission suitability and lengthened transmission seasons. Most significant effects are predicted for parts of Africa, South America, and South-East Asia (51, 52). Within the past 50 years, developing countries had a 39% increase in the number of months suitable for transmission, while effects varied between highland and lowland areas (53, 54). Precipitation patterns have also been shown to influence mosquito survival, as wet areas serve as breeding sites for mosquitos. However, the interaction with precipitation is not yet as clearly understood as it is for temperature (152). Regional decreases in malaria incidence and previously endemic areas becoming unsuitable are also projected in regions where temperatures may exceed optimal transmission temperatures due to the effects of global warming, indicating how climate change may also contribute to fighting malaria in Western Africa and South-East Asia (66). In addition, the expansion of *Anopheles stephensi* to Africa suggests urban transmission will further increase, as it is a competent urban vector with a wider temperature range (153, 154). Prediction models mainly focus on climate variables related to temperature and precipitation and often do not consider indirect factors, such as economic development and improved urban sanitation, the recently approved malaria vaccine, or the increasing resistance to malaria drugs, which may greatly mitigate or exacerbate health outcomes.

*Dengue*, transmitted mainly by mosquitos *Aedes aegypti* and *Aedes albopictus*, is a globally widespread vector-borne disease with rapidly increasing incidence (155). These two mosquitos are experiencing a steady increase in environmental suitability, as indicated by rising transmission rates, with the largest increase seen in countries with a high HDI (53). The risk of dengue fever has shown to be dependent on temperature and precipitation (156, 157). Transmission by *Ae. aegypti* peaks at 29°C, slightly higher than the peak temperature for malaria transmission. Global warming is expected to cause a shift in relative suitability and distribution of these diseases, with the sub-Saharan African region experiencing a shift from malaria to dengue (57). In Africa, where 15.2% of the total continent is already considered a highly suitable area for *Ae. aegypti*, an increase to 21.8% under RCP 4.5 and 23.3% under RCP 8.5 of suitable area is predicted by 2050 (58). An increased suitability for *Ae. albopictus* in Europe was predicted with 83% of urban areas and 68% of the entire European continent being suitable by 2050, up from 49% currently (59).

*Zika and chikungunya viruses* are also transmitted by the *Aedes* mosquito and have the highest burden in the Americas region (158). Their basic reproduction number has increased by around 12% and the length of their transmission season by around 6% compared to the 1950s (62). For the most recent Zika outbreak in 2015 with an estimated 130 million infected people, a link between transmission and El Niño has been suggested (159, 160). A Zika-specific temperature-dependent transmission model for *A. aegypti* predicted a net increase of 2.71 billion people at risk under RCP 8.5, with a projected year-round transmission risk for 915.9 million. Even under RCP 4.5, an additional 2.5 billion people are projected to be at risk as more parts of the world become suitable due to a warming climate. Under this model, the largest increase in transmission suitability was predicted in North America (63).

Chikungunya virus was first identified in Africa, where sporadic cases and outbreaks are reported frequently (161). In recent decades, there have been a few reports of local transmission in Europe (162). As it is transmitted by the same vectors as Zika and dengue, *Ae. aegypti* and *Ae. albopictus*, climate change scenarios project similar expansions due to increased climatic suitability (66, 67). Modeling under RCP 4.5 and RCP 8.5 showed expansion toward regions such as central Europe, China, and Central America; while some areas were also found to have declining suitability (68).

*Yellow Fever* is transmitted by the *Aedes* or *Haemogogus* mosquitos and is endemic in tropical regions of South America and Africa (163). The most recent modeling approach for yellow fever transmitted by *Ae. aegypti* projected an increased transmission intensity and an increase in the number of deaths in the African region, with heterogenous changes across the region, in line with predictions for other vector-borne diseases transmitted by *Ae. aegypti*. An expected increase of the number of deaths per year between 10.0 and 40.0% by 2070, depending on the severity of the anticipated climate change scenario, was reported (70). Meanwhile, another modeling study predicted a decrease in cases and future outbreak durations under RCP 4.5 and RCP 8.5 (71).

*Rift Valley Fever (RVF)* is another mosquito-borne disease of rising concern, a zoonotic, vector-borne disease that causes disease primarily in animals and humans with severe forms such as hemorrhagic fever. Transmission occurs through mosquitos, mainly *Aedes*, and the epidemiology of the virus and its vectors has shown to be influenced by climate; consequently, climate change is predicted to expand vector habitat and contribute to the emergence of risk areas (74, 75). While an increasing risk was associated with ENSO events; rainfall, population density and irrigation have further been identified as environmental drivers, underscoring the need for heightened surveillance efforts (76).

Vector-borne helminth infections are acknowledged as neglected tropical diseases (NTD).

*Lymphatic filariasis* is transmitted by several mosquito species, such as *Anopheles* and *Culex,* whose expanded range and breeding seasons are thought to lead to an estimated 1.86 billion people at risk of infection by 2050 (44, 79). For *Culex*, which also transmits the West Nile Virus, environmental suitability was identified for Europe and North Africa, as well as shifting distribution patterns under different RCP scenarios (80).

*Leishmaniasis* is transmitted by Phlebotomine sandflies, which carry the *Leishmania* parasite. This disease is endemic in many tropical regions; it is climate-sensitive as sandflies are dependent on high temperatures and therefore expected to expand their range under climate change. According to predictions, increased climate suitability could lead to further northward expansion of vector range in Europe; as well as a twofold increase in individuals at risk of Leishmaniasis in North America for 2080 (83, 84).

### 2.3. Tick-borne infections

Ticks are efficient vectors to transmit a wide range of disease-causing pathogens. Important examples are Lyme disease, tick-borne-encephalitis (TBE) and Crimean-Congo Hemorrhagic Fever (CCHF).

*Lyme disease* is the most common tick-borne disease in the United States and Europe (164). The vector of Lyme disease, *Ixodes* ticks, have shown to be sensitive to environmental conditions, foremost temperature, rainfall, and humidity (165). Further, an index based on temperature, precipitation, vegetation, and soil moisture was able to correspond well to observed tick-borne infections, emphasizing the possibility of utilizing these climate variables as surveillance tools (166). Climate change effects are projected to cause an expansion of *Ixodes* distribution and increase the transmission risk, with high regional variability (86–88). Northward expansion of *Ixodes* has already been reported in Northern America and Scandinavia within the last decades, and was attributed to a warming climate in these regions (89, 90). An expansion to higher altitudes has also been projected, along with an increased abundance in regions where ticks are already present (91).

*Tick-borne-encephalitis* is less common than Lyme disease. A shift in distribution to higher altitudes, altered seasonality, and increased incidences were observed in Eastern Europe, as well as increased incidences that were attributed to increasing temperatures in Russia and Sweden (93–97). Furthermore, transmission is predicted to increase further, corresponding to expected temperature increases in Europe (98).

*Crimean-Congo Hemorrhagic Fever (CCHF)* is a disease of major global concern, transmitted by a highly pathogenic virus. Once limited to the 50° North latitude as the geographic range of its vector, CCHF has increased in endemic areas, such as Turkey, and further expanded to northern and western Europe, attributed to a warmer climate (100–102). The principal vector is *Hyalomma* ticks, most commonly *H. marginatum.* Expansion of suitable areas related to changing climatic variables has been observed for *H. marginatum* as well as predictions for further northward expansion with simultaneously reduced suitability in North Africa and Southern Iberia under RCP 4.5 (103, 167).

### 2.4. Air-borne infectious diseases

The effects of climate change on air-borne infectious diseases have been less well studied so far. There is limited evidence suggesting that climate change may influence the dynamics of air-borne infectious diseases. The dynamics of air-borne diseases and their relationship to climate change is complex and warrants further investigation.

*Seasonal influenza*, an acute respiratory infection that occurs in epidemics, is known to be influenced by region-specific climatic factors, foremost temperature and humidity (106, 107). Predictions show a trend where higher temperatures are associated with a lower risk of transmission, while associations with humidity suggest a similar pattern but are less clear (108–110). Consequently, a warming climate with milder winters itself could decrease the risk and intensity of influenza epidemics. However, the climate change-induced weather variability projected under RCP scenarios, with sudden large changes in temperature, may conversely increase the risk of influenza epidemics and result in shifting patterns (111, 112). Extreme weather events, resulting temperature fluctuations and the increasing risk of emerging zoonotic viruses may further contribute to future outbreaks (168). Ultimately, the “net effect” of climate change on influenza, also considering other factors such as vaccination and population density, is difficult to determine. As most current studies focus on temperature and humidity, the influence of other climate-dependent variables that may influence virus survival, replication and mutation should be considered in future studies.

Another important pathogen causing lower respiratory infections is the *Respiratory Syncytial Virus (RSV)*. RSV epidemics are similar to influenza, with temperate regions usually experiencing a peak in winter months. It was found that precipitation and humidity both drive RSV transmission depending on location-specific climate conditions, with increased humidity under RCP 8.5 expected to lead to reduced transmission and precipitation-driven increased transmission under an overall northward shift (115). The role of temperature is less clear and often contradictory. In a study in Canada, warmer temperatures were associated with lower odds of RSV hospitalization (116). In LMICs, the association between temperature and increased or decreased RSV transmission varied by country, so other factors such as humidity and rainfall are likely interwoven (117).

## 3. Prevention and mitigation: the role of vaccines and vaccination

**Figure 1:**
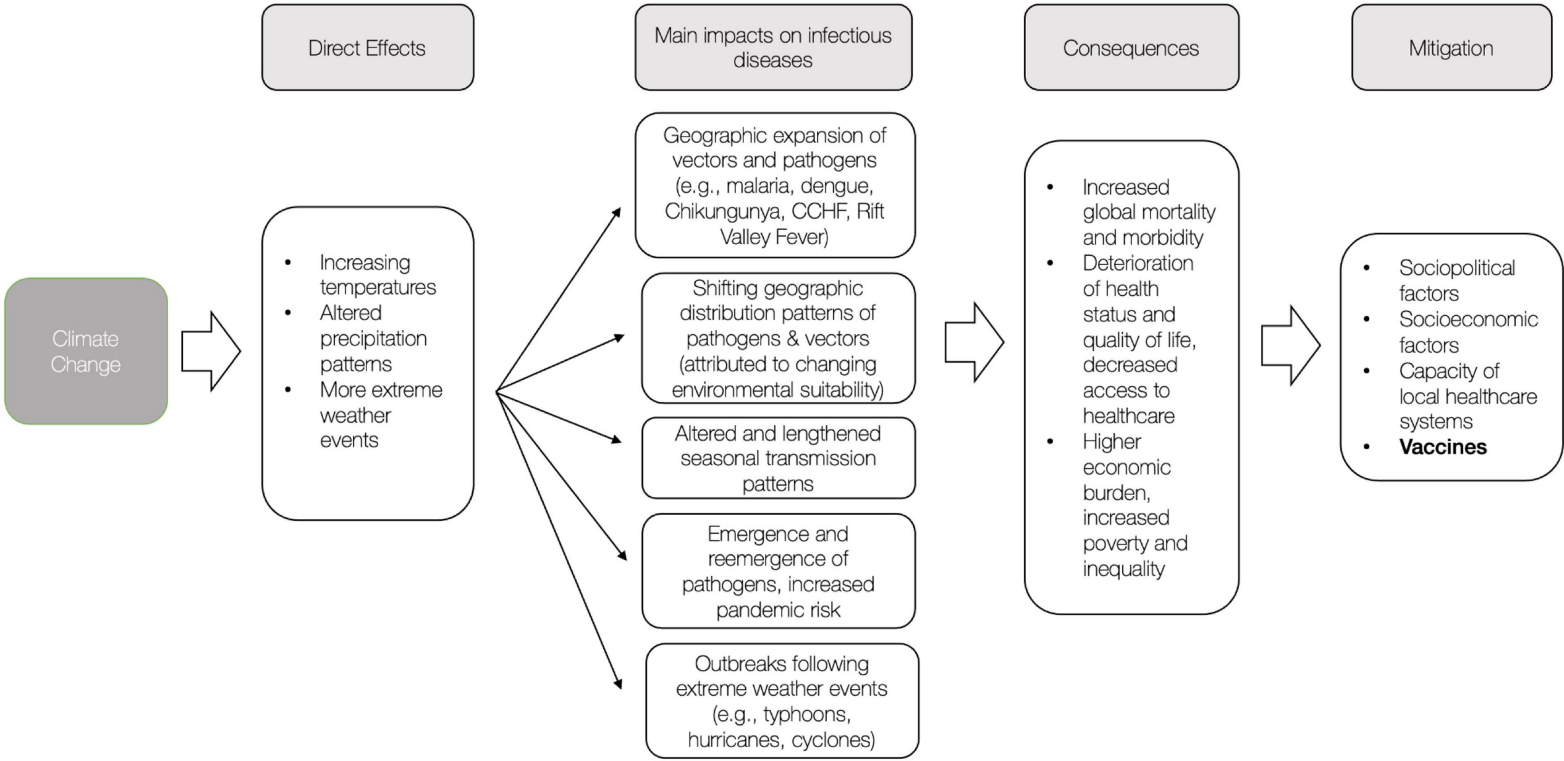
Overview of effects of climate on infectious diseases and mitigation factors.

The next decades will be shaped by the disruptive and destructive consequences of a warming climate, including the exacerbation of climate-sensitive infectious diseases, which poses a major global threat to human health. Figure 1 presents a summary of the impacts and consequences of climate change and subsequent mitigation factors. Taking action to prevent and mitigate potential consequences is critical.

Effective vaccines are highly cost-effective interventions that have substantially reduced the burden of some infectious diseases and eliminated others completely. For some infectious diseases identified in the scope of this review, there are already highly effective vaccines available. These could significantly reduce vulnerability in communities and help protect them from endemic as well as emerging infectious diseases that are driven by climate change. This could lead to strengthened health sector resilience and would help communities to better cope with other impacts of climate change. Further, widespread immunization presents a viable near-term strategy to improve overall health outcomes as opposed to realizing long-term, structural changes for public health in LMICs or combatting the climate crisis itself. In addition, the persisting threat of rising antimicrobial resistance, another major global health threat, could simultaneously be addressed, as vaccines can reduce antibiotic usage and are therefore considered a valuable tool to combat AMR (169).

### 3.1. Cholera vaccine

Cholera illustrates well how an effective vaccine could mitigate the effects of climate change: There are three WHO pre-qualified oral cholera vaccines (OCV): Euvichol-Plus®, Shanchol™, and Dukoral®, which are recommended for use in areas of endemic cholera as well as during outbreaks and humanitarian crises with a high risk of infection (18). These OCVs significantly reduce cholera disease burden, can promote herd immunity, and are cost-effective (170–172). Currently, OCV administration occurs mostly reactively in emergency settings; for this purpose, the global GAVI-funded OCV stockpile has supplied more than 70 million doses for countries in need (173). The WHO announced a critical shortage of cholera vaccines in 2022 due to increased global demand following large outbreaks, which have also been attributed to climate change (174, 175). As the risk of outbreaks further increases, mitigating and preventing them through immunization in communities with poor living conditions, where cholera thrives, could have a substantial impact. Geographic and age-based targeting for vaccination may optimize the use of limited OCV supply by maximizing cost-effectiveness (176, 177). Expanded disease monitoring could help guide national and local vaccine allocation. Resource-limited areas often lack reliable data and diagnostic tools necessary to determine where vaccine distribution is most urgent and will have the greatest benefit (172).

Overcoming current supply constraints in the future could induce a crucial shift from reactive to preventive vaccination with the goal of achieving herd immunity, locally eradicating cholera, and averting epidemic outbreaks altogether. Endemic cholera could potentially be controlled with an estimated 50–70% OCV coverage, emphasizing the substantial benefits of mass vaccination (171). Malawi was one of the first countries to include OCV in their national cholera control plan and administered more than one million doses in the first year in a non-emergency setting, which could serve as a model for other countries (178).

### 3.2. Yellow fever vaccine

Another example of a highly effective vaccine requiring higher coverage is the YF-17D yellow fever vaccine, a one-dose vaccine conferring lifelong protection that has been available since the 1930s (179). National vaccination campaigns have shown to reduce cases and the impact of vaccination significantly influences modeling outcomes for outbreaks (71, 180). However, the actual coverage is estimated at 44% in the African region and has substantially declined within the last decades. Outbreaks continue to occur, and up to 473 million people in risk areas would require vaccination to achieve sufficient population-level coverage (181, 182). There are several second-generation vaccines in development; if shown non-inferior to the YF-17D vaccine, they could provide support to the WHO’s ambitious goal of supplying 1.3 billion doses of vaccines to endemic countries by 2026 and help the global elimination of yellow fever (72, 183).

### 3.3. Malaria vaccine

Developing effective vaccines for vector-borne diseases has proven to be a challenging endeavor that is becoming even more pressing with the climate-induced expansion of vectors. In 2021, after several decades of research and clinical trials, the long-awaited RTS,S/AS01 (Mosquirix™) became the first malaria vaccine and was recommended by the WHO for immunization of children in regions with risk of malaria transmission. More than 2 million doses have been administered in African countries so far (184). However, while it could prevent up to 30% of mortality in young children in endemic regions, efficacy varies between subgroups, is generally modest and protection significantly declines over time. In addition, the four-dose vaccination regime complicates successful implementation in endemic malaria settings (184–188). Weaknesses of the RTS,S vaccine should be considered for further vaccine developments. The R21/Matrix-M vaccine is regarded as a highly promising candidate. It is a modified form of RTS,S, cheaper and more potent, showing efficacy of 77% after the initial doses and up to 80% when administered with a high-dose adjuvant. Phase 3 trials are currently underway with the ambitious goal of obtaining licensure in 2023 (55, 189).

### 3.4. Dengue vaccine

The expansion of dengue is similarly concerning. The only available vaccine CYD-TDV (Dengvaxia®), is licensed in several endemic countries, but its uptake and introduction to routine vaccination has been limited by its variable performance in different age groups, as well as the serotype-dependent response, with seronegative individuals even at higher risk for severe dengue (190). TAK-003 (QDENGA®), a live-attenuated vaccine candidate that is currently being studied in Phase 3 trials, showed high efficacy (80.2%) and reduced hospitalization in infected participants by 95.4% (191). It received a recommendation from the European Medicines Agency in October 2022 and was already approved in Indonesia, which could pave the way for global licensure (192). Effective dengue and malaria vaccines would become an invaluable asset for global public health; considering the projected developments under climate change, even partial protection from vaccines could be substantive and prevent further burden on communities that are already majorly affected.

Infectious diseases expected to be aggravated by climate change and for which no effective preventive vaccine is available yet should be prioritized for research and development (R&D). Limited investment in R&D for poverty-related diseases, which most climate-sensitive diseases currently are, is a fundamental problem expressed by the 10/90 gap. Less than 10% of health R&D expenditure is spent on diseases accounting for more than 90% of the world’s disease burden, the majority of which are infectious diseases. Since the 10/90 gap was first described, global health spending has seen a multifold increase; however, the principle of this gap persists (193). The spending disparities are also reflected in the low number of licensed drugs and vaccines available for these diseases, the result of pharmaceutical and public sector research concentrating on diseases of high-income countries (194). Most of the infectious diseases driven by climate change occur predominantly in developing countries and are therefore a prime example of this gap.

NTDs such as schistosomiasis and soil-transmitted helminths affect hundreds of millions of people and carry a high morbidity, but there are only a few drugs for treatment and no licensed vaccine available yet (195). A few vaccine candidates are currently under development, such as the hookworm recombinant vaccines Na-GST-1/Na-APR-1, in Phase 1 clinical studies (45). There are four prominent candidates with different targets for a Schistosomiasis vaccine that are currently in various clinical phases, including a Phase 1 clinical study for Sm-p80 GLA-SE (SchistoShield®), a leading vaccine candidate (48, 49). Nonetheless, it will take several more years until a licensed NTD vaccine is ready for roll-out and widespread administration, but once available, it has the potential to significantly lessen the burden and improve public health outcomes.

## 4. The way forward: developing vaccines for a changing climate

The IPCC has acknowledged the potential of vaccines to mitigate the effect of climate change on vector-borne disease, along with surveillance and warning systems (3). However, multiple challenges remain to leverage vaccinations as a global strategy to mitigate climate change effects, mainly related to vaccine development, supply, access and delivery.

### 4.1. Adopting climate-responsive vaccine strategies

The first step in climate-responsive strategies should be an improved understanding and anticipation of the dynamic, cascading effects of climate change. Refined spatial and temporal mapping of climate-sensitive diseases would be useful to determine disease burden, which is expected to shift significantly for various diseases; as well as anticipate climate change-fueled extreme weather events that could exacerbate outbreaks of infectious diseases. A proposed “Vaccine Risk Index,” which included variables related to climate, urban population, human development, and peace, could be a valuable tool to identify nations at risk for the emergence and re-emergence of vaccine-preventable diseases and help guide vaccination programs (196).

The climate-related burden assessment would provide further guidance for vaccine development. This review illustrates major gaps in vaccine development for several climate-sensitive diseases, in particular NTDs. Prioritizing research and development for diseases that do not yet have a vaccine and improving less-effective vaccines, while delivering existing vaccines to areas with low coverage should simultaneously be achieved. Research efforts should focus on continuous monitoring of genomic patterns of pathogens, surveillance of resistance to current treatments (e.g., antimicrobial resistance) and current vaccine-induced immune responses in the areas most affected by climate changes. The rapid evolution of SARS-CoV-2 and its variants has led to the development of multivalent (pan sarbecoviruses and zoonotic viruses) vaccines. Such a strategy may be adopted for other rapidly evolving pathogens and the benefit of existing vaccine platforms (e.g., mRNA) for rapid adaptation of vaccine design and development.

The shifting global distribution of diseases warrants a critical assessment of national routine immunization schedules, as some may require future adaptation to include protection against pathogens driven by climate change. In addition, combination vaccines could facilitate vaccine delivery in LMICs by increasing timeliness and efficiency (197). Climate-responsive vaccination strategies should not aspire to take a “one-size-fits-all” approach, rather, they will require dynamic reassessments to determine optimal regional, risk-based recommendations for immunization. Specifically, while vaccines remain a limited resource in several regions of the world, the most vulnerable and at-high-risk populations should be prioritized for vaccinations before large-scale campaigns and routine immunization strategies are implemented.

### 4.2. Pandemic preparedness

The threat of emerging infectious diseases and new pandemics may intensify under climate change (13). Vaccines remain the most effective means of controlling and mitigating outbreaks. The unprecedented, accelerated development of the COVID-19 vaccines with their novel platform technologies and rapid licensing process could serve as a blueprint for future pandemics, while shortcomings of the global roll-out process should be addressed (198). Notably, the United States National Institute of Allergy and Infectious Diseases has recently proposed a blueprint strategy detailing the prototype pathogen approach: developing vaccines directed against known viral families of concern to humans that may be easily and rapidly adapted to a newly identified pathogen and utilized to prevent pandemic outbreaks (199). Rigorous surveillance and disease modeling systems that incorporate anticipated climate change scenarios may help in assessing the expansion of pathogens and vectors, identifying high-risk areas, and predicting the next pandemic.

### 4.3. Scaling up supply and delivery

The recent focus on regional vaccine manufacturing, particularly in Africa, will over the long-term improve availability and accessibility of vaccines in vulnerable communities and LMICs. This will complement the efforts of key global health stakeholders, such as UNICEF and Gavi, The Vaccine Alliance, which currently supply more than 50 low-income countries with low-cost or no-cost vaccines (200–202). Notably, the impact of climate-change-related infectious diseases on populations in LMICs could also encourage the advancement of sustainable, regional vaccine manufacturing. Regardless, vaccines alone are insufficient if vaccination coverage is limited by health sector under-resourcing; most recently, the COVID-19 vaccine roll-out highlighted the importance of strengthening healthcare delivery in LMICs (197, 203). Improving health systems and expanding local and national healthcare infrastructure is crucial to achieve effective vaccine delivery and increase vaccination coverage rates. The global trend of declining vaccination rates, combined with the potential of climate change to undermine the future delivery of vaccines is alarming (204, 205). Vaccines are temperature-sensitive products and must be kept within a strict temperature range throughout their handling until the point of administration. Aware of the logistical challenges of vaccine delivery, considerable efforts are now underway to improve vaccine thermostability and long-term shelf-life at ambient temperature (206, 207). Consequently, implementing robust, climate-resilient vaccine delivery schemes must be a priority. Meanwhile, little is still known about the impact of climate change on host factors that may affect the risk for infectious diseases. For example, a recent study concluded that climate change is likely to affect the susceptibility of individuals to tuberculosis by increasing the prevalence of its underlying risk factors, particularly in LMICs (208). The potential impact of climate change on disease-specific risk host factors warrants further in-depth assessment.

### 4.4. Fighting vaccine hesitancy

Despite the proven safety and efficacy of established vaccines, there is a concerning trend of vaccine hesitancy. This has become increasingly evident during the global roll-out of COVID-19 vaccines and may counter-balance the climate change vaccination mitigation efforts. The WHO has identified vaccine hesitancy as one of the top ten threats to global health (209). Interestingly, in the case of the COVID-19 vaccines, acceptance was shown to be considerably higher for LMICs than high-income countries; this could imply that vaccination strategies should not be exclusive to LMICs but must also focus on high-income countries to overcome regional and national vaccination coverage gaps (210). Further, vaccine hesitancy has enabled resurgences of vaccine-preventable diseases such as measles, where even a small decrease in vaccine coverage could lead to significant increases in disease incidence (211).

Ultimately, the investment in vaccines will yield multiple beneficial downstream effects: these include the reduction of global disease burden, inequalities, poverty, antimicrobial resistance and potential future pandemics, as well as the improvement in quality of life, economic growth and planetary health; overall contributing to achieving the United Nation’s Sustainable Development Goals and ensuring health in a warming world (212).

## 5. Conclusion

The climate change crisis is undeniable and will be the ultimate challenge of the 21st century. Though still limited, evidence for the impact of climate change on infectious disease is accumulating. The impact of climate change on infectious diseases is confounded by interrelated covariates, including, but not limited to, human mitigation measures, geographic distribution, and variations within countries. Important non-climate determinants of disease transmission, such as globalization, public health systems, and socioeconomic conditions, may also be affected indirectly by climate change and should be addressed simultaneously.

### 5.1. Prevention and mitigation

Further research and monitoring in the form of large-scale, longitudinal studies are needed to better understand the health impacts of climate and environmental changes as they are constantly evolving. Meanwhile, collaborative surveillance activities are also essential measures to improve predictive models and outbreak preparedness, while guiding global and local policymaking for the introduction and prioritization of vaccination schemes.

### 5.2. Developing vaccines to mitigate the impact of climate change

Currently available vaccines should be distributed to achieve wider coverage, especially in LMICs. Whether single vaccines for specific diseases or combined “climate change” vaccines should be developed for better use are questions that deserve to be addressed urgently by stakeholders and developers. Considering the potentially severe effects of climate change on infectious diseases, health sector resilience should be strengthened. In this context, global vaccination strategies may serve as an important mitigation tool and must become a priority for research and development, supply, and delivery.

## Author contributions

J-LE and JK designed the synopsis. CK and SA wrote the manuscript. FM, JK, and J-LE reviewed and edited the manuscript. All authors contributed to the article and approved the submitted version.
